# Evaluation of the cytotoxic and immunogenic potential of temozolamide, panobinostat, and *Lophophora williamsii* extract against C6 glioma cells

**DOI:** 10.17179/excli2020-3181

**Published:** 2021-03-09

**Authors:** Moisés Armides Franco-Molina, Silvia Elena Santana-Krímskaya, Luis Mario Madrigal-de-León, Erika Evangelina Coronado-Cerda, Diana Ginette Zárate-Triviño, Sara Paola Hernández-Martínez, Paola Leonor García-Coronado, Cristina Rodríguez-Padilla

**Affiliations:** 1 Universidad Autónoma de Nuevo León (UANL), Facultad de Ciencias Biológicas, Laboratorio de Inmunología y Virología, P.O. Box 46 "F", 66455, San Nicolás de los Garza, NL, México; 2 Universidad del Valle de México, Campus Cumbres, Departamento de Ciencias de la Salud, Av. Las Palmas, 5500, Colonia Cima de las Cumbres, Monterrey, Nuevo León, C.P. 64610, Mexico

**Keywords:** Lophophora williamsii, temozolamide, panobinostat, glioblastoma, immunogenic cell death, DAMPs

## Abstract

Glioblastoma multiforme is a malignant neoplasm of the brain with poor prognosis. The first-line drug against glioblastoma is the alkylating agent temozolamide (TMZ); unfortunately, treatment resistance and tumor re-incidence are common. In some cases, immunogenic cell death (ICD) inducers can decrease treatment resistance and tumor recurrence by stimulating an antitumor specific immune response. Not all ICD inducers, however, are suitable for glioma patients because of the low permeability of the blood-brain barrier (BBB). Panobinostat (PAN), a histone deacetylase inhibitor and *Lophophora williamsii (LW) *extract can pass through the BBB and have antitumor properties. The aim of this study is to evaluate the cytotoxic potential of TMZ, PAN and *LW *extract against the glioma C6 cell line, and its role in the release of damage-associated molecular patterns (DAMPs), which is a hallmark of ICD. Our results indicate that all treatments induce cellular death in a time- and concentration-dependent manner, and that PAN and *LW *extract induce apoptosis, whereas TMZ induces apoptosis and necrosis. Also, that some of the treatments and their sequential administration induce the release of DAMPs. Furthermore, in a rat glioma model, we observed that all treatments decreased tumor volume, but the *in vivo* cell death mechanism was not ICD. Our findings indicate that TMZ, PAN, and *LW *combination have a cytotoxic effect against glioma cells but do not induce ICD.

## Abbreviations

**TMZ** temozolamide

**PAN** panobinostat

***LW*** *Lophophora williamsii*

**ICD** immunogenic cell death

**BBB** blood-brain barrier

**DAMPs** damage-associated molecular patterns

## Introduction

Glioblastoma multiforme (GBM) is malignant neoplasia of the brain that originates in the glial cells within the intracranial tissue. GBM infiltrates the surrounding tissue and decreases neurological function, resulting in a poor quality of life for the patients (Bahadur et al., 2019[3]). Current therapies against GBM involve a combination of surgery, chemotherapy, and radiotherapy, nevertheless, it has a median overall survival of 2 years (Singleton et al., 2017[16]). 

The alkylating agent temozolamide (TMZ) is a first-line drug for the treatment of glioblastoma; it acts by damaging tumor cells through DNA methylation, changing heterochromatin organization, and activating an oxidative stress response. However, tumor cells quickly become resistant to TMZ at pharmacotherapeutic concentrations (Stepanenko et al., 2016[17]).

Some therapeutic drugs can induce immunogenic cell death (ICD) in cancer cells. ICD originates with endoplasmic reticulum stress in the target cell that results in an elevated production of reactive oxygen species and the release of damage-associated molecular patterns (DAMPs). The release of DAMPs includes the exposure of calreticulin (CRT) and the extracellular release of ATP, high-mobility group box 1 protein (HMGB1), and heat shock proteins (HSP70 and HSP90) (Turubanova et al., 2019[19]). Once released, DAMPs interact with innate immune cell receptors, which then become activated. Activated innate immune cells interact with lymphocytes to generate an adaptative antitumor immune response that hinders cancer relapse (Rapoport and Anderson, 2019[13]; Du and Waxman, 2020[7]). The most frequently used ICD inducers would not clinically benefit glioma patients due to the low permeability of the blood-brain barrier (BBB).

Effective treatment for glioblastoma requires substances able to cross the BBB. One of these substances is panobinostat (PAN), an epigenetic modulator that inhibits histone deacetylase activity and increases DNA-histone acetylation. This blocks multiple signals related to the development and progress of tumors, and induces apoptosis in the target cells (Van Veggel et al., 2018[20]). Furthermore, increased histone acetylation sensitizes cancer cells to the effect of alkylating agents, such as TMZ (Stiborova et al., 2012[18]).

*Lophophora williamsii* (*LW*) also known as “peyote”, is a spineless cactus known for the physical, visual, and perceptual changes it induces upon ingestion (Casado et al., 2008[4]). *LW* extract has immunostimulatory properties: it increases macrophage cytokine production and lymphocyte proliferation, and it is cytotoxic for cancer cell lines (Alonso-Castro et al., 2016[1]). *LW* extract also crosses the BBB (Dinis-Oliveira et al., 2019[6]) and some of its components interact with the serotonergic 5-HT2A-C receptors present in glioblastoma cells (Lu et al., 2020[10]). 

The use of multiple treatments for cancer has been of great importance to improve therapeutic outcome. The present study was designed to determine the antitumor effect of TMZ, PAN, and *LW* extract on a rat glioma model, and their capacity to induce ICD. 

## Materials and Methods

### Reagents

Temozolomide (TMZ) was purchased from Schering-Plough (Kenilworth, NJ, USA), panobinostat (PAN) was purchased from Cellagen Technology (San Diego, CA, USA), and the methanolic extract of *Lophophora williamsii *(*LW*) was obtained from a plant collection of the Laboratorio de Inmunología y Virología, Facultad de Ciencias Biológicas, UANL. IgG_1 _mouse HSP70 antibody (sc-32239), IgG_1 _mouse HMGB1 antibody (sc-56698), IgG_2a _mouse HSP90α/β (sc-13119), and IgGκ mouse HRP antibody (sc-516102) were purchased from Santa Cruz Biotechnology (Dallas, TX, USA). Dulbecco´s Modified Eagle Medium (DMEM) was purchased from Sigma-Aldrich (St. Louis, MO, USA).

### Preparation of Lophophora williamsii extract 

The cacti used in this study belonged to a plant collection of the Laboratorio de Inmunología y Virología, Facultad de Ciencias Biológicas, UANL, and had been previously identified as *Lophophora williamsii*. The cacti were macerated and methanol extraction was performed for 48 h at 4 °C, after which the ethanolic phase was filtered. The methanol extract was lyophilized using a freeze-dryer (Labconco Co. Kansas City, MO). The resulting powder was dissolved in 1 mL of DMEM and the endotoxin levels were measured with the gel clot-based Limulus amoebocyte assay (Associates of Cape Cod. Falmouth, MA), which has a detection limit of 0.004 ng/mL.

### Cell line and culture conditions

The C6 murine brain glial cell line was purchased from the American Type Culture Collection (Manassas, Virginia, USA). Cells were cultured in DMEM supplemented with bovine fetal serum (10 % v/v) in a 5 % CO_2_ atmosphere at 37 °C.

### Cytotoxicity assay

C6 cells (5x10^3^) were seeded into 96-well plates and cultured overnight in a 5 % CO_2_ atmosphere at 37 °C. Then, cells were treated with TMZ (2.15 - 43 mM), and *LW *extract (1.44 - 4.8 mg/mL) for 24 and 48 h, and with PAN (0.5 - 25 *µ*M) for 24, 48 and 72 h. Also, the TMZ + *LW *extract + PAN treatment combination was evaluated using the IC_30_, and IC_50 _for 24 h. After the treatment, cells were washed with phosphate-buffered saline (PBS) 1X, and cellular metabolic activity was assessed using the resazurin assay. Cells were incubated with a resazurin solution (20 % v/v) for 30 min in a 5 % CO_2_ atmosphere at 37 °C and the fluorescence was measured using a microplate reader Synergy TM HT (BioTek Instrument, Vermont, NH, USA) at 535/590 nm, excitation/emission wavelength, respectively. The percentage of cell viability (%) was calculated according to the following equation [1]: 

Relative viability (%) = treated cell fluorescence ÷ control cell fluorescence x 100 [1]

### Chemosensitivity assay

C6 cells (5x10^3^) were seeded into 96-well plates and cultured overnight in a 5 % CO_2_ atmosphere at 37 °C. Then cells were pre-exposed to TMZ (2.15 - 43 mM) for 24 h, followed by the treatments with *LW* extract (1.92 mg/mL, IC_50_ value) and PAN (0.75 *µ*M, IC_50_ value) for 24 h; pre-exposure to PAN (0.5 - 25 *µ*M) for 24 h, followed by the treatments with *LW* extract (1.92 mg/mL, IC_50_ value) and TMZ (8.6 mM, IC_50_ value) for 24; and pre-exposure to *LW* extract (1.44 - 4.8 mg/mL) for 24 h, followed by PAN (0.75 *µ*M, IC_50_ value) and TMZ (8.6 mM, IC_50_ value) treatments for 24 h. Finally, cells were washed with PBS 1X and cell metabolic activity was assessed using the resazurin assay as previously described. 

### Recovery assay

C6 cells (5x10^3^) were seeded into 96-well plates and cultured overnight in a 5 % CO_2 _atmosphere at 37 °C. After this, cells were treated with TMZ (2.15 - 43 mM), and *LW* extract (1.94 - 4.8 mg/mL) for 24 h, and with PAN (0.5 - 25 *µ*M) for 24 and 72 h. The treatments were removed, and cells were washed twice with PBS 1X. Then DMEM supplemented with bovine fetal serum (10 % v/v) was added and cells were maintained at a 5 % CO_2_ and 37 °C atmosphere for 5 days. Finally, cells were washed with PBS 1X and cell metabolic activity was assessed using the resazurin assay. 

### Apoptosis and cell viability assay

Acridine orange/ethidium bromide (AO/EB) staining was used to determine viable and nonviable cells, based on disrupted cell membrane. Cells (1x10^5^) were seeded in 6 well plates and treated with TMZ (IC_50_ = 8.6 and IC_100_ = 43 mM), and *LW* extract (IC_50_ = 1.92 and IC_100_ = 4.8 mg/mL) for 24 h, and PAN (IC_50_ = 0.75 and IC_100_ = 25 *µ*M) for 72 h. Also, cells pre-exposed to TMZ (8.6 mM) for 24 h followed by *LW* extract (1.92 mg/mL) and PAN (0.75 *µ*M) 24 h treatments, were included. Briefly, cells were washed with PBS 1X and stained with 20 µL of AO/EB dye mix (100 *µ*L/mL AO and 100 *µ*L/mL EB, prepared in PBS). Then wells were observed under a confocal fluorescence microscope (Olympus X70), at 250/605 nm (excitation/ emission wavelength, respectively) for EB and 502/525 nm (excitation/emission wavelength, respectively) for AO. Viable cells were identified by bright green fluorescence, apoptotic cells by bright orange fluorescence, and necrotic cells by bright red fluorescence.

### Determination of DAMPs

The production of DAMPs was detected by indirect ELISA assay in cell lysates and supernatants after treatment with TMZ, *LW* extract, and PAN. 

C6 cells (5x10^6^) were treated with the IC_100_ of TMZ (43 mM), *LW* extract (4.8 mg/mL), PAN (25 µM), or pre-exposed to TMZ (43 mM) for 24 h, followed by *LW* extract (4.8 mg/mL) and PAN (25 *µ*M) treatments for 24 h. After the treatments, the cells were collected and centrifuged at 1,200 rpm for 10 min at room temperature. Then cells were washed with PBS 1X and homogenized using the SET 2X lysis buffer (20 mM Tris pH 6.8, 2 mM EDTA pH 8.0, 300 mM NaCl and 4 % SDS; Sigma-Aldrich: Merck) supplemented with complete Halt protease inhibitor cocktail. Protein quantification was performed using the BCA kit (cat. No. 23225, Pierce^TM^ BCA Protein Assay Kit, Thermo Fisher, Waltham, Massachusetts, USA). 

To perform the indirect ELISA assay, 96-well plates were pre-coated with 25 µg of protein from each sample for 24 h at 4 °C. After this, a specific primary antibody for HMGB1, HSP70, or HSP90 was added to the plates and incubated for 2 h at 37 °C. To detect the presence of DAMPs a mouse IgG HRP-conjugated secondary antibody was added to each well and incubated for 30 min at room temperature. Then, a chromogen solution was added, and the plates were incubated at room temperature for 30 min. Finally, the reaction was terminated by the addition of a stop solution, and absorbance was determined at a 450 nm wavelength. 

For the detection of DAMPs in the supernatant, C6 cells (5x10^6^) were treated with the IC_100_ of TMZ, *LW* extract, and PAN for 24 h, or a pre-exposure treatment with TMZ (43 mM) for 24 h, followed by *LW* extract (4.8 mg/mL) and PAN (25 *µ*M) for 24 h. After this, the supernatants were collected and HMGB1, HSP70, and HSP90 were determined as described above.

### Animals

Data were obtained from 2- to 3-months-old female Wistar rats with a weight range of 240-260 g. The animals (n=25) were provided by the bioterium of the Facultad de Ciencias Biológicas, UANL. Rats were kept in 12 h light/dark cycles with *ad libitum* water and food. All animal procedures were performed according to the Official Mexican Norm of Animal Welfare NOM-033-SAG/ZOO-2014 and approved by the internal Research and Animal Welfare Ethics Committee (CEIBA) of the Facultad de Ciencias Biológicas, UANL (2018-015).

### C6 cells inoculation and whole tumor cell lysate vaccination 

A total of 6x10^6^ C6 cells were treated *in vitro* with TMZ (43 mM), *LW* extract (4.8 mg/mL), or PAN (25 *µ*M) for 24 h, and a pre-exposure treatment with TMZ (8.6 mM) for 24 h, followed by *LW* extract (1.92 mg/mL) and PAN (0.75 *µ*M) combination for 24 h. Next, the cells were centrifuged at 1,200 rpm for 10 min and washed twice with PBS 1X. Finally, cells were resuspended in 300 *µ*L of PBS 1X and inoculated subcutaneously into the left flank of Wistar rats. The rats were randomly divided into five experimental groups: 1) Control group: without vaccination (n = 5), 2) TMZ group (n = 5), 3) *LW* extract group (n = 5), 4) PAN group (n = 5), and 5) pre-exposure treatment with TMZ followed by *LW *extract + PAN group (n = 5). After 7 days, rats were challenged with 5x10^6 ^viable C6 cells resuspended in 300 *µ*L of PBS 1X via subcutaneous injection into the left flank. Tumor width and length were measured every third day for 21 days with a digital caliper and tumor volume was calculated according to the formula: V = (W^2 x L)/2, where V is tumor volume, W is tumor width and L is tumor length (Santana-Krímskaya et al., 2020[15]).

The humane endpoint was used to avoid unnecessary suffering, and rats were sacrificed 21 days after inoculation.

### Statistical analysis

The experiments were performed in triplicate and statistical differences between groups were analyzed using ANOVA followed by the Tukey post hoc test. The data are presented as mean ± standard deviation (SD) and differences between groups were considered significant at a *p-value* ≤ 0.05. Statistical analyses were performed with the GraphPad Prism software version 6 (GraphPad Software, Inc., San Diego, Ca).

## Results

### Treatments with TMZ, LW extract, and PAN decreased C6 cells viability in a concentration-dependent manner

**Figure 1:**
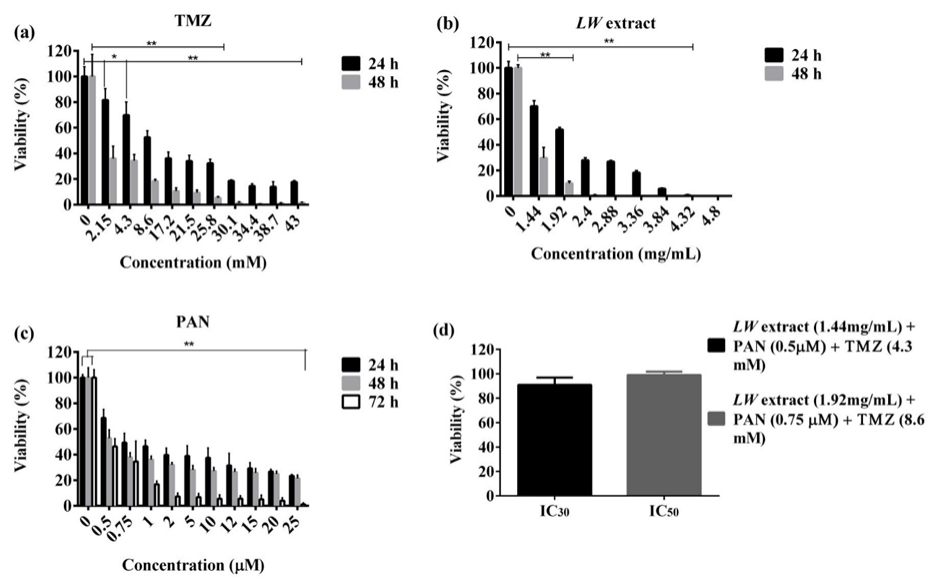
TMZ, *LW* extract, and PAN induce cell death in C6 cells as assessed by the resazurin assay. (a) Representative bar graphs of data obtained of viable cells treated at 24 and 48 h with TMZ. (b) Representative bar graphs of data obtained of viable cells treated at 24, and 48 h with *LW* extract. (c) Representative bar graphs of data obtained of viable cells treated at 24, 48, and 72 h with PAN. (d) Representative bar graphs of data obtained of viable cells treated with a combination of IC_30_, and IC_50_ of all treatments at 24 h. Results were obtained from three independent experiments performed in triplicate, and data are presented as the mean ± standard deviation. **p = 0.001 and *p = 0.01 with respect to control. TMZ, temozolomide; *LW* extract, *Lophophora williamsii* extract; PAN, panobinostat

The TMZ and *LW* extract treatments significantly reduced the viability of C6 cells (*p *< 0.001) as compared to untreated cells in a time- and concentration-dependent manner. The IC_50_ value was 8.6 mM for TMZ, and 1.92 mg/mL for *LW* extract at 24 h (Figure 1a and 1b(Fig. 1)). In the case of PAN, we observed a 50 % reduction of viability at the 0.75 *µ*M concentration after 24 h of treatment, and a significant cytotoxic effect (*p* < 0.001) was observed after 48 and 72 h with all concentrations of PAN used (Figure 1c(Fig. 1)). In contrast, the treatment combination TMZ + *LW* extract + PAN was not cytotoxic at the IC_30_, or IC_50 _values (Figure 1d(Fig. 1)).

### Pre-exposure treatment enhanced the cytotoxic effect against C6 cells

**Figure 2:**
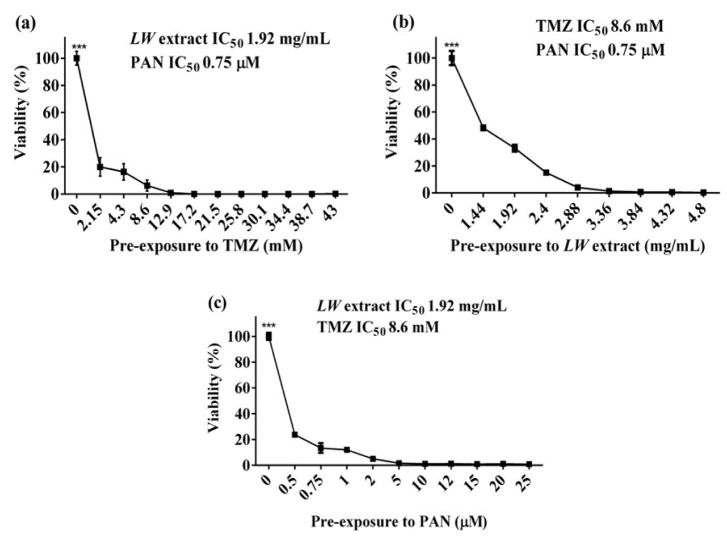
Pre-exposure to individual treatments for 24 h increases the cytotoxic effect over C6 cells as assessed by the resazurin assay. (a) Pre-exposure with TMZ for 24 h, followed by *LW* extract (1.92 mg/mL), and PAN (0.75 µM) for 24 h. (b) Pre-exposure with *LW* extract for 24 h, followed by TMZ (8.6 mM), and PAN (0.75 µM) for 24 h. (c) Pre-exposure with PAN for 24 h, followed by IC_50 _value of *LW* extract (1.92 mg/mL), and TMZ (8.6 mM) for 24 h. Results were obtained from three independent experiments performed in triplicate, and data are presented as the mean ± standard deviation. ***p = 0.0001 with respect to control. TMZ, temozolomide; *LW* extract, *Lophophora williamsii* extract; PAN, panobinostat

Pre-exposure with TMZ for 24 h followed by treatment with a combination of PAN and *LW* extract at IC_50_ values (0.75 *µ*M and 1.92 mg/mL, respectively) increased significantly (*p *< 0.0001) the cytotoxic effect of TMZ against C6 cells (Figure 2a(Fig. 2)). 

Similarly, *LW* extract pre-exposure for 24 h followed by PAN (0.75 *µ*M) and TMZ (8.6 mM) combination treatment (Figure 2b(Fig. 2)), and PAN pre-exposure treatment for 24 h, followed by *LW* extract (1.92 mg/mL) and TMZ (8.6 mM) (Figure 2c(Fig. 2)), also increased the cytotoxic effect.

### TMZ, LW extract, and PAN treatments prevent the recovery of C6 cells

**Figure 3:**
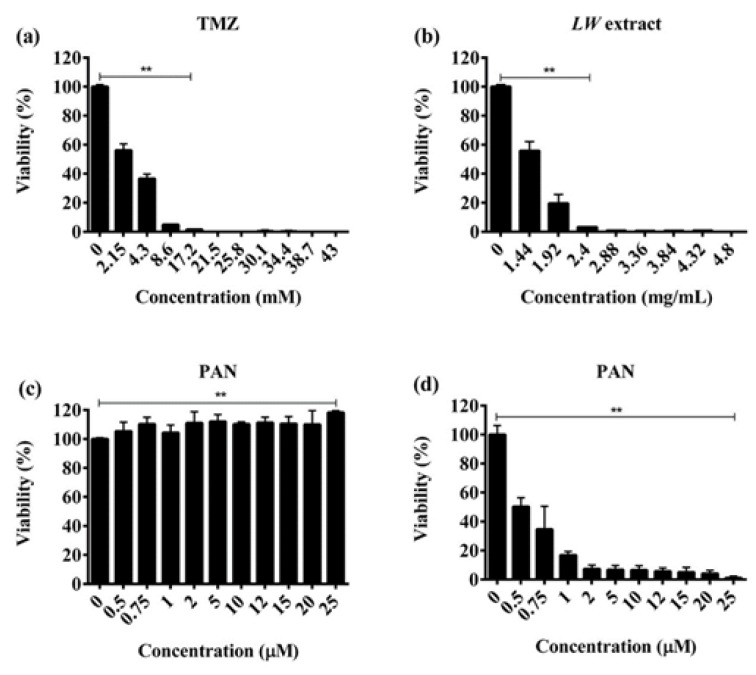
TMZ, *LW* extract, and PAN avoid the recovery of C6 cells as assessed by the resazurin assay. (a) Viability of C6 cells 24 h after removing the TMZ for a recovery period of 5 days. (b) Viability of C6 cells 24 h after removing the *LW* extract for a recovery period of 5 days. (c) Viability of C6 cells 24 h after removing the PAN for a recovery period of 5 days. (d) Viability of C6 cells 72 h after removing the PAN for a recovery period of 5 days. Results were obtained from three independent experiments performed in triplicate, and data are presented as the mean ± standard deviation. **p = 0.001 with respect to the control. TMZ, temozolomide; *LW *extract, *Lophophora williamsii* extract; PAN, panobinostat

To determine the recuperation capacity of C6 cells after the treatments, cell viability was measured after a recovery period of 5 days (Figure 3(Fig. 3)). Treatment with TMZ and *LW *extract significantly (*p* < 0.001) decreased cell recovery of C6 cells in a dose-dependent manner (Figure 3a and 3b(Fig. 3)). The PAN treatment significantly (*p* < 0.001) decreased cell recovery in a time- and dose-dependent manner (Figure 3c and 3d(Fig. 3)).

### TMZ, LW extract, and PAN treatments induce cell death by apoptosis and necrosis on C6 cells

**Figure 4:**
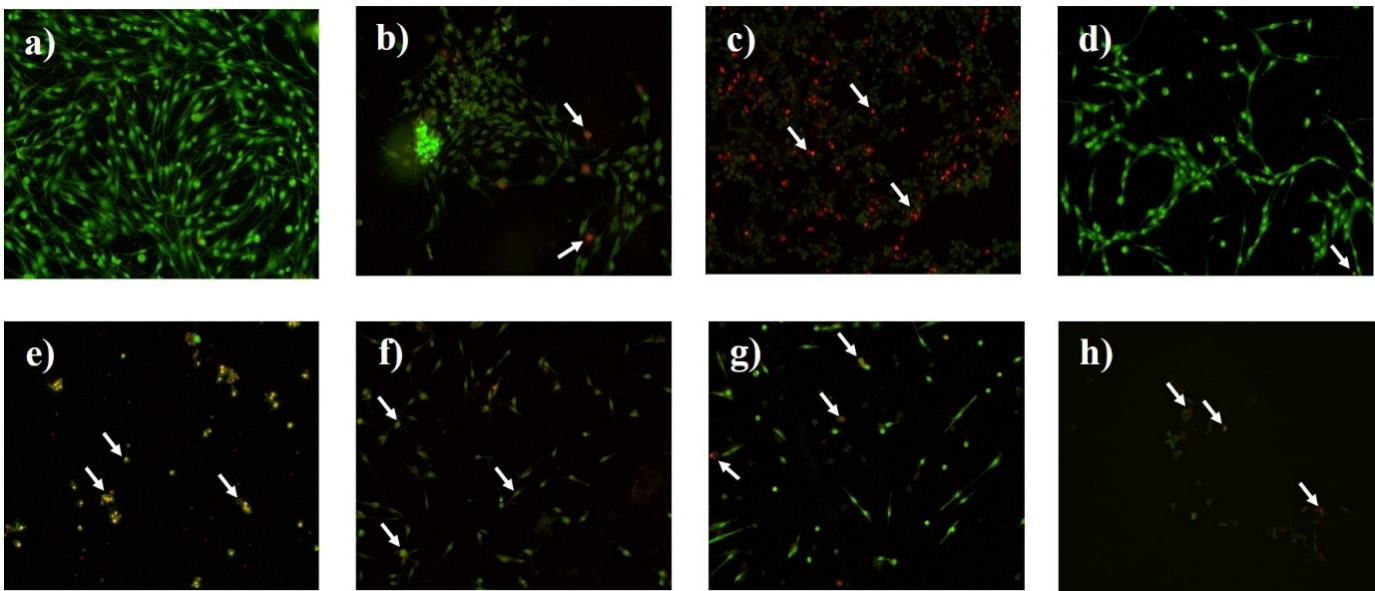
AO/EB staining assay shows that treatments TMZ, *LW* extract, and PAN induce apoptosis and necrosis on C6 cells. The arrows indicate cell damage. (a) Nontreated cells. (b) Cells treated with the IC_50_ of TMZ (8.6 mM) for 24 h. (c) Cells treated with the IC_100_ of TMZ (43 mM) for 24 h. (d) Cells treated with the IC_50 _of *LW* extract (1.92 mg/mL) for 24 h. (e) Cells treated with the IC_100_ of *LW* extract (4.8 mg/mL) for 24 h. (f) Cells treated with the IC_50_ of PAN (0.75 µM) for 72 h. (g) Cells treated with the IC_100_ of PAN (25 µM) for 72 h. (h) Cells pre-exposed to TMZ (8.6 mM) for 24 h followed by *LW* extract (1.92 mg/mL) and PAN (0.75 µM). TMZ, temozolomide; *LW* extract, *Lophophora williamsii* extract; PAN, panobinostat

Untreated cells presented a bright green nuclear fluorescence and a regular structure (Figure 4a(Fig. 4)). Cells treated with the IC_50_ of TMZ showed early stages of apoptosis (bright orange cell fluorescence) (Figure 4b(Fig. 4)), and cells treated with the IC_100 _showed necrosis (bright red cell fluorescence) (Figure 4c(Fig. 4)). The IC_50_ and IC_100 _of* LW* extract (Figure 4d(Fig. 4)), induced cell death by apoptosis (Figure 4e(Fig. 4)), as did the IC_50_ and IC_100_ of PAN (Figure 4f and 4g(Fig. 4)). Finally, TMZ pre-exposition followed by *LW* extract and PAN treatments induced cell death by necrosis (Figure 4h(Fig. 4)).

### TMZ, PAN, and pre-exposure to TMZ followed by LW extract and PAN increase the release of HMGB1, HSP70, and HSP90 by C6 cells

**Figure 5:**
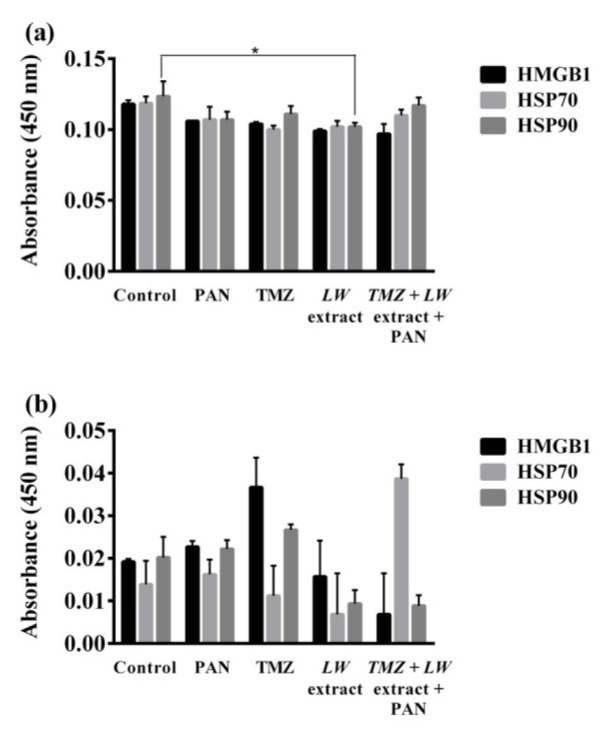
TMZ, PAN, and TMZ followed by *LW* extract and PAN stimulate the release of HMGB1, HSP70, and HSP90 as measured by ELISA. (a) HMGB1, HSP70, and HSP90 measurement in cell lysates after 24 h of treatment. (b) HMGB1, HSP70, and HSP90 measurement in the supernatant after 24 h of treatment. Results were obtained from three independent experiments performed in triplicate, and data are presented as the mean ± standard deviation. *p = 0.01 with respect to the control. TMZ, temozolomide; *LW* extract, *Lophophora williamsii* extract; PAN, panobinostat; HMGB1, high-mobility group box 1 protein, HSP70 and HSP90, heat shock protein 70 and 90, respectively

There were no significant differences between the levels of HMGB1, HSP70, and HSP90 found in cell lysates, however, there was a significant (*p *= 0.01) difference in the HSP90 levels of *LW* treated cells and the control (Figure 5a(Fig. 5)). In the supernatant, TMZ and PAN treatments increased the release of HMBG1 and HSP90 as compared to the control, but this increase was not significant (Figure 5b(Fig. 5)). On the other hand, the pre-exposure to TMZ followed by *LW* extract and PAN treatment increased the release of HSP70, in contrast to the control, but there was no statistical significance (Figure 5b(Fig. 5)).

### TMZ, LW extract, PAN and TMZ pre-exposure followed by LW extract and PAN do not induce immunogenic cell death in vivo

**Figure 6:**
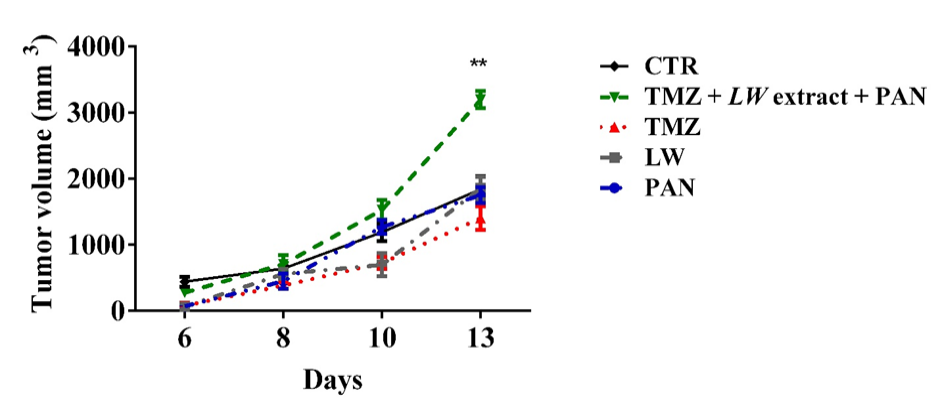
TMZ, *LW* extract, PAN, and TMZ and followed by *LW *extract and PAN did not induce immunogenic cell death. The tumor volume was measured every third day for 13 days starting at day 7 post-inoculation. Results are presented as the mean ± standard deviation. **p = 0.001. TMZ, temozolomide; *LW* extract, *Lophophora williamsii* extract; PAN, panobinostat

C6 cells lysed by TMZ, *LW* extract, PAN, or pre-exposure to TMZ followed by *LW* extract and PAN treatments were used to vaccinate rats, which were then challenged with viable C6 cells. Tumor presence was detected at day 6 in all rats (vaccinated and unvaccinated). The tumor volume was measured until day 13. The group vaccinated with the cell lysed by pre-exposition to TMZ and treated with *LW* extract and PAN presented the highest tumor volume, compared to the other groups (*p* = 0.001) (Figure 6(Fig. 6) and Supplementary Tables 1 to 6).

## Discussion

The present study aimed to determine the cytotoxic and immunogenic potential of TMZ, PAN, and *LW* extract in a C6 glioma rat model. 

Our results show that the cytotoxic effect of all tested treatments (TMZ, PAN, and *LW* extract) decreased C6 cell viability in a time- and concentration-dependent manner (Ni et al., 2019[12]). To our knowledge, there are no previous reports of the cytotoxic effect of PAN against C6 cells, but it has been reported that other histone-modifying enzymes (BIX01294, 3DZNep, VP, TSA, and chaetocin) do not affect C6 cells viability after 72 hours of exposure (Maleszewska et al., 2014[11]). The difference is that this group did not include PAN, for which the apoptotic effect had been previously described, although not against glioma cells (Gerson et al., 2018[9]). Regarding the *LW* extract cytotoxic effect, our research group had previously reported that *LW* methanolic extract decreases the viability of murine fibrosarcoma L929, murine lymphoma L5178Y-R, human histiocytic lymphoma U937, and human breast cancer MCF7 (Franco-Molina et al., 2003[8]) cell lines. However, there are no previous reports of the effect of *LW* extract over C6 or other glioma cell lines.

The recovery assay allowed us to discriminate cell death from a temporarily inactive metabolism. TMZ, PAN, and *LW *extract treated cells were unable to proliferate even after a five-day recovery period, corroborating cell death.

The single treatments induced apoptosis, but the simultaneous combination of all treatments did not exert any cytotoxic effect. The sequential treatment, which consisted of C6 cells pre-exposition to TMZ followed by treatment with *LW* extract and PAN resulted in necrosis. The combination of cytotoxic drugs tends to cause antagonism, but this antagonism does not necessarily translate into clinical failure because of other properties, such as immune response modulation, which can also inhibit tumor growth (Richards et al., 2020[14]). The use of multiple agents can result in the release of DAMPs from dying cells, and these molecules interact with immune cells to trigger an antitumor adaptive immune response (Zhou et al., 2019[22]; Asadzadeh et al., 2020[2]). 

In this study, we did not observe a significant increase of HMGB1 and HSP90 in the supernatant of C6 cells treated with TMZ (8.6 mM) and PAN (20 µM) as compared to the control, but there was an increase of HSP70 in cells pre-exposed to TMZ, followed by *LW* extract and PAN.

Finally, we evaluated ICD induction *in vivo*. The treatments did not prevent the implantation of the C6 glioma, suggesting that immunogenic death was not the *in vivo *cell death mechanism. These results correlate with the recurrence of human glioma after a few months following completion of TMZ treatment (Daniel et al., 2019[5]), indicating the lack of a tumor-specific immune response. Furthermore, it has been reported that TMZ treatment upregulates programmed cell death-1 ligand-1 (PD-L1) in glioblastoma cells, promoting immune escape (Wang et al., 2019[21]). If this is the case, PD-L1 expression could be antagonizing the effect of DAMPs release. 

## Conclusions

In conclusion, this study contributes to the knowledge that TMZ, PAN, or *LW* extract and its combinations do not induce immunogenic cell death, suggesting an explanation regarding the tumor recurrence in glioma patients after conventional treatment; furthermore, the use of *LW* extract should be considered for glioma treatment, alone or sequentially administrated with TMZ and PAN, but further *in vivo* experiments should be performed to determine its antitumor effect.

## Funding

“Fondo Sectorial de Investigación para la Educación”, grant A1-S-35951, CONACYT, México supported this work.

## Acknowledgements

We thank Alejandra Elizabeth Arreola Triana for article revision; and Universidad Autónoma de Nuevo León UANL, Facultad de Ciencias Biológicas, Laboratorio de Inmunología y Virología, P.O. Box 46 “F”, 66455 San Nicolás de los Garza, NL, México for facilities provided. 

## Conflicts of interest

The authors declare that there is no conflict of interest.

## Supplementary Material

Supplementary data
