# Supplementary material for: Evaluation of the cytotoxic and immunogenic potential of temozolamide, panobinostat, and Lophophora williamsii extract against C6 glioma cells
Source: EXCLI J. 2021 Mar 9;20:614–24. doi: 10.17179/excli2020-3181 (PMC8056056; doi:10.17179/excli2020-3181)
Supplement: Supplementary data [file EXCLI-20-614-s-001.pdf]

EXCLI Journal 2021;20:614-624 – ISSN 1611-2156
Received: November 18, 2020, accepted: February 15, 2021, published: March 09, 2021

Supplementary data to:

Original article:

EVALUATION OF THE CYTOTOXIC AND IMMUNOGENIC POTENTIAL OF TEMOZOLAMIDE, PANOBINOSTAT, AND *LOPHOPHORA WILLIAMSII* EXTRACT AGAINST C6 GLIOMA CELLS

Moisés Armides Franco-Molina[1,*], Silvia Elena Santana-Krímskaya[1], Luis Mario Madrigal-de-León[1], Erika Evangelina Coronado-Cerda[2], Diana Ginette Zárate-Triviño[1], Sara Paola Hernández-Martínez[1], Paola Leonor García-Coronado[1], Cristina Rodríguez-Padilla[1]

1 Universidad Autónoma de Nuevo León (UANL), Facultad de Ciencias Biológicas, Laboratorio de Inmunología y Virología, P.O. Box 46 “F”, 66455, San Nicolás de los Garza, NL, México
2 Universidad del Valle de México, Campus Cumbres, Departamento de Ciencias de la Salud, Av. Las Palmas, 5500, Colonia Cima de las Cumbres, Monterrey, Nuevo León, C.P. 64610, Mexico

* **Corresponding author:** Dr. Moisés Armides Franco-Molina, Universidad Autónoma de Nuevo León (UANL), Facultad de Ciencias Biológicas, Laboratorio de Inmunología y Virología, P.O. Box 46 “F”, 66455, San Nicolás de los Garza, NL, México.
Tel.: +52-812-141-15, extension: 6428;
E-mail: moyfranco@gmail.com, moises.francoml@uanl.edu.mx

http://dx.doi.org/10.17179/excli2020-3181

This is an Open Access article distributed under the terms of the Creative Commons Attribution License (http://creativecommons.org/licenses/by/4.0/).

EXCLI Journal 2021;20:614-624 – ISSN 1611-2156
Received: November 18, 2020, accepted: February 15, 2021, published: March 09, 2021

Supplementary Table 1 (raw data to Figure 6 in main text): Tumor volume (mm^3) of rats vaccinated with tumor cell lysates of C6 cells. The tumor volume was measured every third day for 13 days starting at day 7 post-inoculation. Results are presented as the mean ± standard deviation. $^{**}p = 0.001$. CRT, control; TMZ, temozolomide; *LW* extract, *Lophophora williamsii* extract; PAN, panobinostat

	Groups				
Day	CRT (n=5)	TMZ (n=5)	PAN (n=5)	LW extract (n=5)	TMZ + LW extract + PAN (n=5)
6	440.0 ± 76.4	80.3 ± 39.4	72.9 ± 29.3	65.8 ± 24.5	277.1 ± 22.6
8	592.0 ± 36.9	383.9 ± 58.4	448.0 ± 112.4	564.7 ± 174.2	722.7 ± 99.3
10	1010.0 ± 134.1	723.9 ± 77.9	1277.1 ± 114.8	1277.1 ± 114.8	1537.6 ± 138.8
13	1533.9 ± 188.1	1408.4 ± 167.2	1750.7 ± 126.5	1788.6 ± 189.4	3184.9 ± 125.6**

Supplementary Table 2 (raw data to Figure 6 in main text): Tumor volume (mm^3) raw data of unvaccinated rats. The tumor volume was measured every third day for 13 days starting at day 7 post-inoculation. The tumor volume of each unvaccinated rat is presented, as well as the mean and standard deviation (SD) per day

	Control group						
Day	animal no. 1	animal no. 2	animal no. 3	animal no. 4	animal no. 5	mean	SD
6	440.0	400.0	550.0	480.0	350.0	444.0	76.4
8	592.0	633.0	660.0	626.0	690.0	640.2	36.9
10	1010.0	1300.0	1095.0	1225.0	1321.0	1190.2	134.1
13	1533.9	1910.5	2024.3	1932.4	1820.6	1844.3	188.1

Supplementary Table 3 (raw data to Figure 6 in main text): Tumor volume (mm^3) raw data of TMZ lysate vaccinated rats. The tumor volume was measured every third day for 13 days starting at day 7 post-inoculation. The tumor volume of each rat vaccinated with TMZ lysed C6 cells is presented as well as the mean and standard deviation (SD) per day. TMZ, temozolomide

	TMZ group						
Day	animal no. 1	animal no. 2	animal no. 3	animal no. 4	animal no. 5	mean	SD
6	89.6	65.2	63.1	40.2	143.4	80.3	39.4
8	310.1	424.4	459.2	365.6	360.2	383.9	58.4
10	790.3	806.2	630.5	734.2	658.5	723.9	77.9
13	1620.2	1543.6	1222.3	1305.1	1350.6	1408.4	167.2

EXCLI Journal 2021;20:614-624 – ISSN 1611-2156
Received: November 18, 2020, accepted: February 15, 2021, published: March 09, 2021

Supplementary Table 4 (raw data to Figure 6 in main text): Tumor volume (mm^3) raw data of PAN lysate vaccinated rats. The tumor volume was measured every third day for 13 days starting at day 7 post-inoculation. The tumor volume of each rat vaccinated with PAN lysed C6 cells is presented as well as the mean and standard deviation (SD) per day. PAN, panobinostat

PAN group

Day	animal no. 1	animal no. 2	animal no. 3	animal no. 4	animal no. 5	mean	SD
**6**	35.1	94.4	109.2	65.6	60.2	72.9	29.3
**8**	519.1	300.3	404.3	422.8	593.4	448.0	112.4
**10**	1255.4	1393.2	1125.2	1389.6	1222.2	1277.1	114.8
**13**	1825.5	1799.3	1620.4	1614.4	1893.8	1750.7	126.5

Supplementary Table 5 (raw data to Figure 6 in main text): Tumor volume (mm^3) raw data of *LW* extract lysate vaccinated rats. The tumor volume was measured every third day for 13 days starting at day 7 post-inoculation. The tumor volume of each rat vaccinated with *LW* extract lysed C6 cells is presented as well as the mean and standard deviation (SD) per day. *LW* extract, *Lophophora williamsii* extract

***LW* extract group**

Day	animal no. 1	animal no. 2	animal no. 3	animal no. 4	animal no. 5	mean	SD
**6**	72.9	97.6	29.2	65.6	63.7	65.8	24.5
**8**	416.8	690.3	654.3	722.5	339.4	564.7	174.2
**10**	1255.4	1393.2	1125.2	1389.6	1222.2	1277.1	114.8
**13**	1835.9	1500.4	2012.7	1859.4	1734.5	1788.6	189.4

Supplementary Table 6 (raw data to Figure 6 in main text): Tumor volume (mm^3) raw data of TMZ + *LW* extract + PAN lysate vaccinated rats. The tumor volume was measured every third day for 13 days starting at day 7 post-inoculation. The tumor volume of each rat vaccinated with TMZ + *LW* extract + PAN lysed C6 cells is presented as well as the mean and standard deviation (SD) per day. TMZ, temozolomide; PAN, panobinostat; *LW* extract, *Lophophora williamsii extract*

TMZ + *LW* extract + PAN group

Day	animal no. 1	animal no. 2	animal no. 3	animal no. 4	animal no. 5	mean	SD
**6**	273.1	297.8	302.9	252.9	258.6	277.1	22.6
**8**	792.1	735.7	556.1	725.3	804.5	722.7	99.3
**10**	1432.7	1609.1	1734.6	1389.6	1522.2	1537.6	138.8
**13**	3187.5	3102.4	3032.5	3245.6	3356.4	3184.9	125.6
